# Pore geometry control of apparent wetting in porous media

**DOI:** 10.1038/s41598-018-34146-8

**Published:** 2018-10-24

**Authors:** Harris Sajjad Rabbani, Benzhong Zhao, Ruben Juanes, Nima Shokri

**Affiliations:** 1 School of Chemical Engineering and Analytical Science, The University of Manchester, Manchester, M13 9PL United Kingdom; 2 Department of Civil Engineering, McMaster University, Hamilton, ON Canada; 3 Department of Civil and Environmental Engineering, Massachusetts Institute of Technology, Cambridge, Massachusetts USA; 4 Department of Earth, Atmospheric and Planetary Sciences, Massachusetts Institute of Technology, Cambridge, Massachusetts USA

## Abstract

Wettability, or preferential affinity of a fluid to a solid substrate in the presence of another fluid, plays a critical role in the statics and dynamics of fluid-fluid displacement in porous media. The complex confined geometry of porous media, however, makes upscaling of microscopic wettability to the macroscale a nontrivial task. Here, we elucidate the contribution of pore geometry in controlling the apparent wettability characteristics of a porous medium. Using direct numerical simulations of fluid-fluid displacement, we study the reversal of interface curvature in a single converging-diverging capillary, and demonstrate the co-existence of concave and convex interfaces in a porous medium—a phenomenon that we also observe in laboratory micromodel experiments. We show that under intermediate contact angles the sign of interface curvature is strongly influenced by the pore geometry. We capture the interplay between surface chemical properties and pore geometry in the form of a dimensionless quantity, the apparent wettability number, which predicts the conditions under which concave and convex interfaces co-exist. Our findings advance the fundamental understanding of wettability in confined geometries, with implications to macroscopic multiphase-flow processes in porous media, from fuel cells to enhanced oil recovery.

## Introduction

Wettability is the preferential affinity of a fluid with the solid surface in the presence of another immiscible fluid^1–3^, and it plays a crucial role in the distribution of fluid phases in the pore space^4–11^. Wettability is typically characterized by the static contact angle *θ*, measured between a smooth horizontal solid surface and the fluid-fluid interface^2^. Perfectly wetting systems (*θ* = 0) and partially wetting systems (*θ* > 0) exhibit fundamentally different behaviour in terms of liquid spreading^3^—in this study we are concerned exclusively with partially wetting systems. We adopt the convention that *θ* is measured through the defending phase (the fluid phase being displaced), and we will use the term *wetting* (to the defending phase) when *θ* < 90°, *nonwetting* (to the defending phase) when *θ* > 90°, and *neutrally wetting* for the particular case *θ* = 90°. From the assumption of local phase equilibrium, the relationship between *θ* and surface forces is described by Young’s law^2^:

1 $$\sigma \,\cos \,\theta =\,{\sigma }_{if}-{\sigma }_{df},$$

where *σ*, *σ*_*if*_ and *σ*_*df*_ are the interfacial energies of the fluid-fluid, invading fluid-solid, and, defending fluid-solid interfaces, respectively. According to Young’s law, the wetting case (*θ* < 90°) corresponds to *σ*_*if*_ > *σ*_*df*_, and the nonwetting case (*θ* > 90°) to *σ*_*if*_ < *σ*_*df*_. In the former case, the surface has higher affinity for the defending fluid, while in the latter case it has higher affinity for the invading fluid^2^.

Many previous studies have demonstrated the impact of wettability on different aspects of multiphase flow in porous media^4–22^. An important question, however, is how the *effective* wettability of a porous medium emerges from the surface wettability at the microscale (as determined by the contact angle *θ*). Early considerations^23^ suggest that contact angle alone provides an incomplete description, and that accounting for pore geometry is necessary to arrive at a more predictive description of the *apparent wettability* of a porous medium, which multiphase flow dynamics so critically depend on.

Despite the importance of apparent wettability on multiphase flow in porous media, to date only few recent studies have directly measured the local wetting characteristics in porous media^9,24–29^, and a predictive theoretical framework linking the effect of surface wettability and pore geometry is missing. Here, we investigate the interplay of surface wettability and pore geometry in determining the apparent wetting characteristics of a porous medium. We do so by first performing direct numerical simulations of fluid-fluid displacement in a single channel of variable cross section, which allows us to assess and extend earlier descriptions of capillary pressure on converging-diverging capillaries^23^. We then study, via numerical simulations, the impact of pore geometry on the slow displacement of one fluid by another in a 2D porous medium under different surface wettability conditions, and demonstrate its profound control on macroscopic features of the displacement. We uncover the coexistence of convex and concave fluid-fluid interfaces at intermediate wettabilities—a feature that has remained absent from existing theories of multiphase flow in porous media—and provide experimental evidence of such convex-concave coexistence from direct imaging of immiscible fluid-fluid displacements in patterned microfluidic cells. Finally, we propose a simple relationship between the apparent wettability of the porous medium and the combined effect of surface wettability and pore geometry, which in turn can serve as a diagnostic tool for macroscale predictions.

## Fluid-Fluid Displacement In Single Capillaries

### Simulation setup

**Figure 1:**
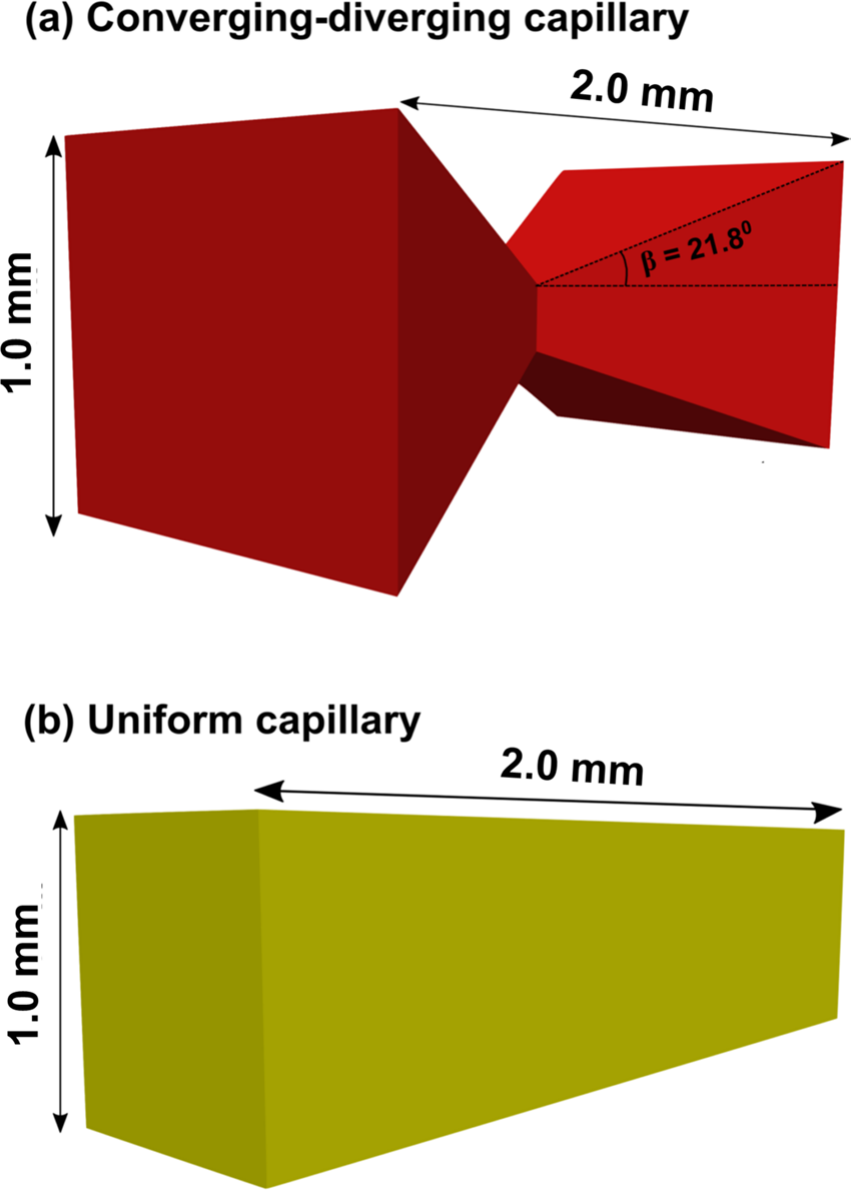
3D representation of the capillaries used in the numerical simulations. (**a**) Converging-diverging geometry. (**b**) Uniform square cross-section.

We perform Computational Fluid Dynamics (CFD) simulations, where we solve the Navier-Stokes equations coupled with the volume of fluid method using a finite volume approach. The details of the numerical formulation are described elsewhere^30^, and will not be repeated here. We simulate fluid-fluid displacement in two capillaries with square cross-section: one with a uniform cross section, and the other with a non-uniform (converging-diverging) geometry (Fig. 1).

The non-uniform capillary converges and diverges at an angle of orientation *β* = 21.8°, and the length of the capillaries was kept constant at 2 × 10^−3^ m. The capillaries are initially filled with the defending fluid (viscosity *μ*_*d*_ = 1.0 × 10^−3^ Pa · s and density *ρ*_*d*_ = 1000 kg · m^−3^), which is displaced by the invading fluid (viscosity *μ*_*i*_ = 1.0 × 10^−3^ Pa · s, density *ρ*_*i*_ = 1000 kg · m^−3^) at a constant flow rate *Q* = 7.0 × 10^−12^ m^3^ · s^−1^. The interfacial tension between the fluids is *σ* = 70 × 10^−3^ N · m^−1^. The outlet of the capillaries is kept at atmospheric pressure in all simulations. The static contact angle *θ*, which corresponds to the angle between the defending fluid and the solid surface, is defined as an input parameter in the solver. The numerical simulations are performed at three different *θ* values: 45° (drainage), 90° (neutral wetting) and 135° (imbibition). The simulations do not take into account contact angle hysteresis and dynamic contact angle effects. The relative importance of viscous to capillary forces is characterised using the capillary number ${\rm{Ca}}=\frac{{\mu }_{i}{v}_{i}}{\sigma }$, where *v*_*i*_ is the injection velocity of the invading fluid^31^. All simulations are performed at Ca = 10^−7^, a value for which capillary forces dominate viscous forces. Gravity forces do not play a role in the simulations since the fluid densities are identical. The visualization and post-processing of the numerical results are performed using ParaView^32^.

### Curvature reversal of the interface in a converging-diverging capillary

**Figure 2:**
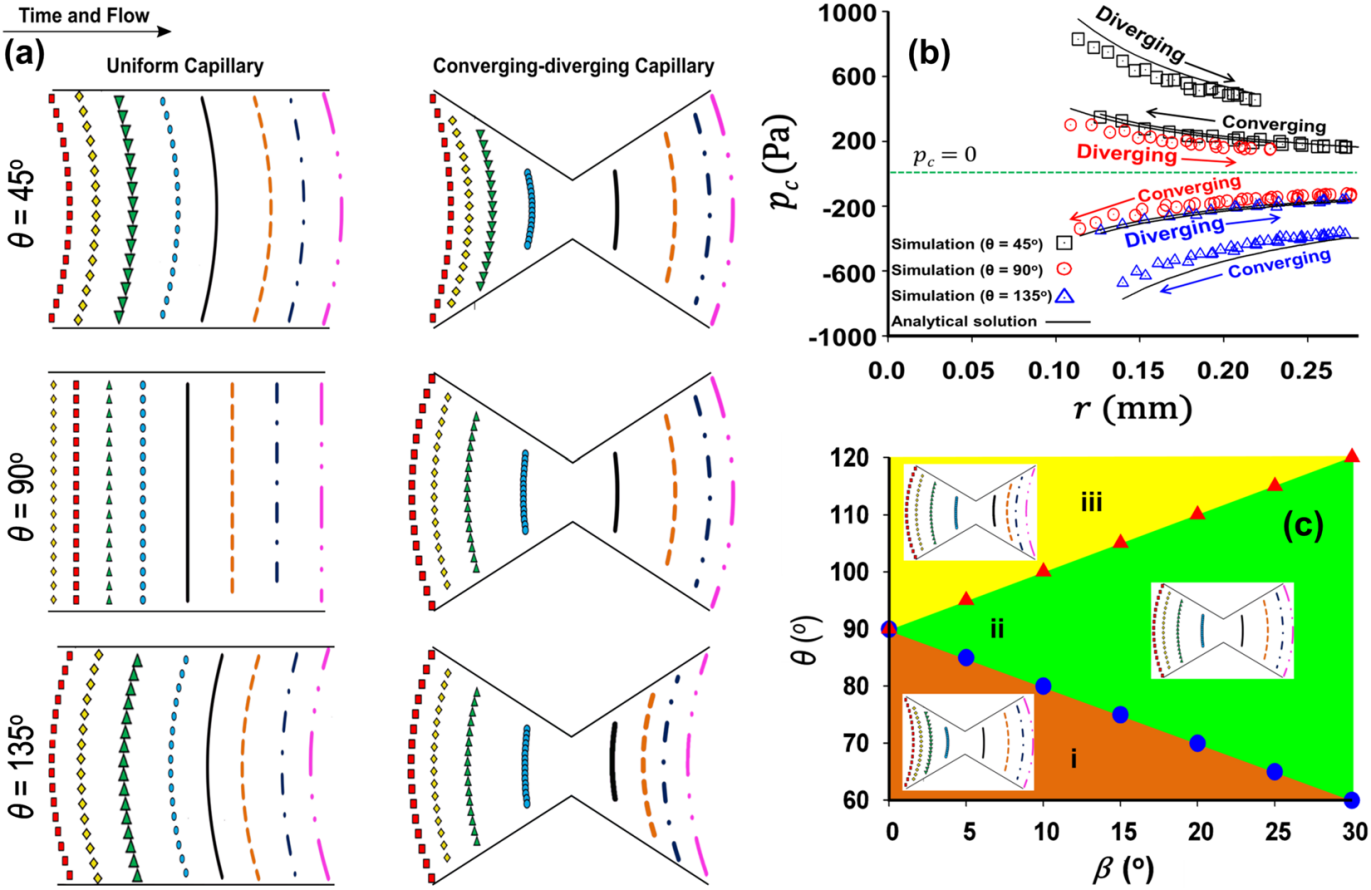
(**a**) Movement of the fluid-fluid interface in uniform and converging-diverging capillaries at different contact angles *θ*. Colour lines denote the position of the interface at the vertical plane of symmetry of the 3D capillaries (see Fig. 1). In the uniform capillary, the interface is simply translated, maintaining its shape and curvature for all values of *θ*. In the non-uniform capillary, in contrast, the shape of the interface changes between the converging section and the diverging section of the capillary. This change is particularly notable for *θ* = 90°: there is a transition in the interface curvature from concave to convex as the interface moves from the converging to the diverging section. (**b**) Evolution of capillary pressure *p*_*c*_ (Pa) as a function of the local capillary radius *r*, as the interface moves from the converging to the diverging section of the capillary, for different values of *θ*. In drainage (*θ* = 45°, black squares), the interface is convex along the entire capillary, and *p*_*c*_ > 0. In imbibition (*θ* = 135°, blue triangles), the interface is concave throughout, and *p*_*c*_ < 0. In the neutral wetting case (*θ* = 90°, red circles), in contrast, the interface is concave in the converging section (*p*_*c*_ < 0), and convex in the diverging section (*p*_*c*_ > 0), illustrating the contribution of the capillary angle *β* in modulating *p*_*c*_. (**c**) Phase-diagram in *θ*–*β* space, separating the region where the apparent wettability switches from wetting to nonwetting as a result of pore geometry ((ii) shaded green), to the regions that remain uniformly wetting ((i) shaded brown) and nonwetting ((iii) shaded yellow). The boundaries between regions correspond to the values for which the interface is flat in the converging section (*θ* = *π*/2 − *β*, blue circles) and in the diverging section (*θ* = *π*/2 + *β*, red triangles).

The movement of the interface in the uniform and converging-diverging capillary for different values of the contact angle *θ* is shown in Fig. 2(a). To provide a quantitative analysis of interface morphology in the converging-diverging capillary, variations in capillary pressure *p*_*c*_ as a function of the local capillary radius *r*(radius of the capillary at the contact line) are presented in Fig. 2(b). The Laplace pressure, or capillary pressure *p*_*c*_, represents the pressure difference across the interface, *p*_*c*_ ≡ *p*_*i*_ − *p*_*d*_, where *p*_*i*_ is the pressure of the invading fluid and *p*_*d*_ is the pressure of the defending fluid, and is given by the Laplace equation

2 $${p}_{c}=\sigma \kappa ,$$

where *κ* is the curvature of the interface, computed as the median value of the curvature distribution from the diffuse interface produced by the numerical solution^30^.

Figure 2(a,b) show that for *θ* = 135° or 45° the sign of *p*_*c*_ does not change as the interface traverses the converging-diverging capillary with *β* = 21.8°, and the convexity of the interface is the same in the uniform and non-uniform capillaries. For *θ* = 45°, the invading fluid is the non-wetting fluid (convex interface and positive *p*_*c*_) and the displacement process is regarded as drainage. For *θ* = 135°, the invading fluid is the wetting fluid (concave interface and negative *p*_*c*_) and the displacement process is regarded as imbibition. In contrast, for *θ* = 90°, there is a difference in the morphology of the interface in the uniform and converging-diverging capillaries. In the uniform capillary, the interface is flat, and *p*_*c*_ = 0. In the converging-diverging capillary, however, the interface flips from concave to convex (Fig. 2(a)), so that *p*_*c*_ changes sign as the interface moves from the converging to the diverging section (Fig. 2(b)).

Understanding the sign of *κ* as an indicator of apparent wettability^23^ provides a way to rationalize the effect of (microscopic) surface wettability and pore geometry on the wettability characteristics of porous media. Figure 2(a,b) shows that under strong drainage ($\theta \ll 90^\circ$) and under strong imbibition ($\theta \gg 90^\circ$) the sign of the fluid-fluid interface curvature coincides with that of cos *θ* in Young’s equation [Eq. (1)], and remains unchanged as the interface traverses the converging-diverging pore. In the case of near-neutral wetting (*θ* ~ 90°), however, the orientation angle *β* exerts a strong control over the apparent wettability: the invading fluid *imbibes* in the converging section (*p*_*c*_ < 0) and *drains* defending fluid in the diverging section of the capillary (*p*_*c*_ > 0).

To gain more insight into the phenomenon of curvature reversal in the converging-diverging capillary, we have obtained an analytical model relating the capillary pressure *p*_*c*_ with static contact angle and the geometric characteristics of the pore. Following Rabbani *et al*.^30^ (Eq. (14)), and replacing cos *θ* by cos(*θ* ± *β*), one obtains the analytical expression describing the capillary pressure in the converging (+) and diverging (−) sections of the capillary:

3 $${p}_{c}=\frac{2\pi r\sigma }{G{P}^{2}}(1-\frac{h}{2\pi })\frac{\sin (\pi -h)}{\sin (h/2)}\,\cos (\theta \pm \beta ),$$

where *h* is the corner angle of the capillary cross-section, *P* is the perimeter of the capillary cross-section, and *G* = *C*/*P*^2^ is the so-called shape factor, where *C* is the cross-sectional area of the capillary.

The comparison between the CFD simulation results and the above analytical solution is shown in Fig. 2(b). It is apparent from Eq. (3) that it is the algebraic sum of *θ* and *β* that controls the sign of interface curvature *κ*, as suggested in previous studies^17,23,33^. Indeed, the equation cos(*θ* ± *β*) = *π*/2 determines the boundaries of the phase diagram in *θ*–*β* space (Fig. 2(c)), separating the region where the apparent wettability switches from wetting to nonwetting as a result of pore geometry (ii), to the regions that remain uniformly wetting (i) and nonwetting (iii). Later, we upscale Eq. (3) to derive an effective, or apparent, wettability number that takes into account the chemical affinity of the solid to the fluids, as well as the geometric characteristics of the porous medium.

## Fluid-Fluid Displacement In 2D Micromodels

### Description of the 2D flow micromodel

**Figure 3:**
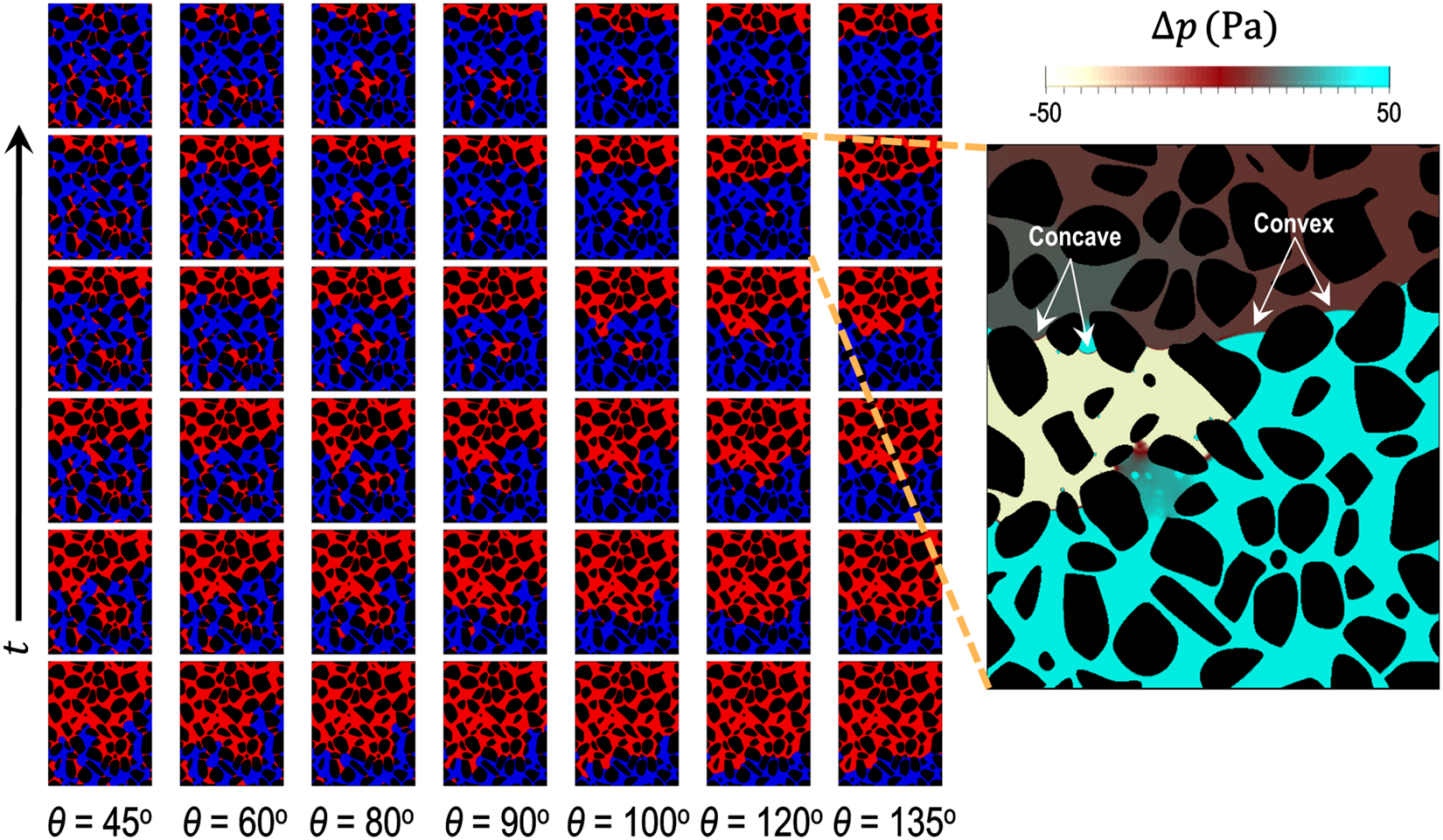
Time evolution of the simulated displacement pattern as a function of the static contact angle *θ* in the 2D micromodel. The direction of flow is from bottom to top. Blue, red and black indicate the invading fluid, the defending fluid and the grains, respectively. The magnified image shows a map of the pressure drop Δ*p* = *p* − *p*_*o*_ where *p* is the pressure at each point and *p*_*o*_ is the outlet pressure, for the case *θ* = 120°. This magnified image indicates that there is a co-existence of concave and convex interfaces, which stems from curvature reversal at the converging and diverging sections of the pore throat.

We simulate immiscible fluid-fluid displacements in a 2D porous medium or micromodel to investigate the macroscopic consequences of geometrically induced appparent wetting. The design of the 2D micromodel (see Fig. S1 in the Supplemental Information) is based on a cross-section of a real sand pack obtained by 3D X-ray micro-tomography^34^. The pore size distribution of the micromodel ranges from 0.028 to 0.308 mm with an average pore size *r*_*p*_ of 0.12 mm. The pore size distribution was obtained by first applying the MATLAB (Image Processing Toolbox) function ‘bwdist’ on the binary image to compute the distance between each pixel in the image and the nearest boundary. Subsequently, the MATLAB function ‘bwmorph’ was used to retain only the center pixel of each pore. The fluid properties and boundary conditions employed in the simulation are similar to those used for the simulations in the single capillary, except for the changes to *μ*_*d*_ = 10^−1^ Pa · s and Ca = 10^−5^. The static contact angle *θ* imposed at the grain boundaries is uniform throughout the micromodel. Figure 3(a) illustrates typical examples of phase distributions in the micromodel at intermediate contact angles *θ* ranging from 45° to 135°.

### Co-existence of concave and convex interfaces in a porous medium

**Figure 4:**
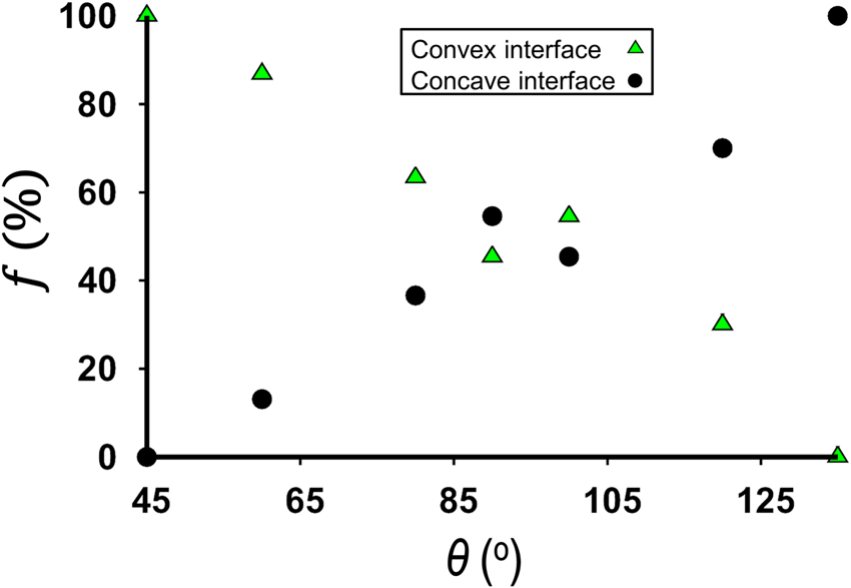
Fraction 𝑓 (%) of concave and convex interfaces when the displacement front is approximately 8 mm away from the intlet. Overall, the fraction of concave interface increases while the fraction of convex interfaces decreases as *θ* is increased from 45° to 135°. In the range of *θ* between 60° and 120°, substantial co-existence of concave and convex interfaces occurs.

Figure 3 shows the co-existence of concave and convex interfaces despite the micromodel having a uniform static contact angle of *θ* = 120°. The co-existence of the concave and convex interfaces is a consequence of curvature reversal from converging sections to diverging sections of a pore-throat, similar to that observed in the converging-diverging capillary (Fig. 2). Consistent with this observation, the pressure field in the invading fluid for *θ* = 120° indicates that drainage (convex interface) and imbibition (concave interface) are taking place simultaneously, even in neighboring pores (Fig. 3, inset). This behavior, which is reminiscent of a fractional-wet system, demonstrates that the local apparent wettability depends on the pore geometry. To probe the impact of pore geometry on the local apparent wettability at different static contact angles, we plot the fraction 𝑓 (%) of concave interfaces (against the total of concave and convex interfaces) as a function of *θ* (Fig. 4).

Figure 4 shows that only concave interfaces (i.e. imbibition) are present at *θ* = 135°, while only convex interfaces are present (i.e. drainage) at θ = 45°. The co-existence of concave and convex interfaces is observed for *θ* ranging from approximately 60° to 120°.

**Figure 5:**
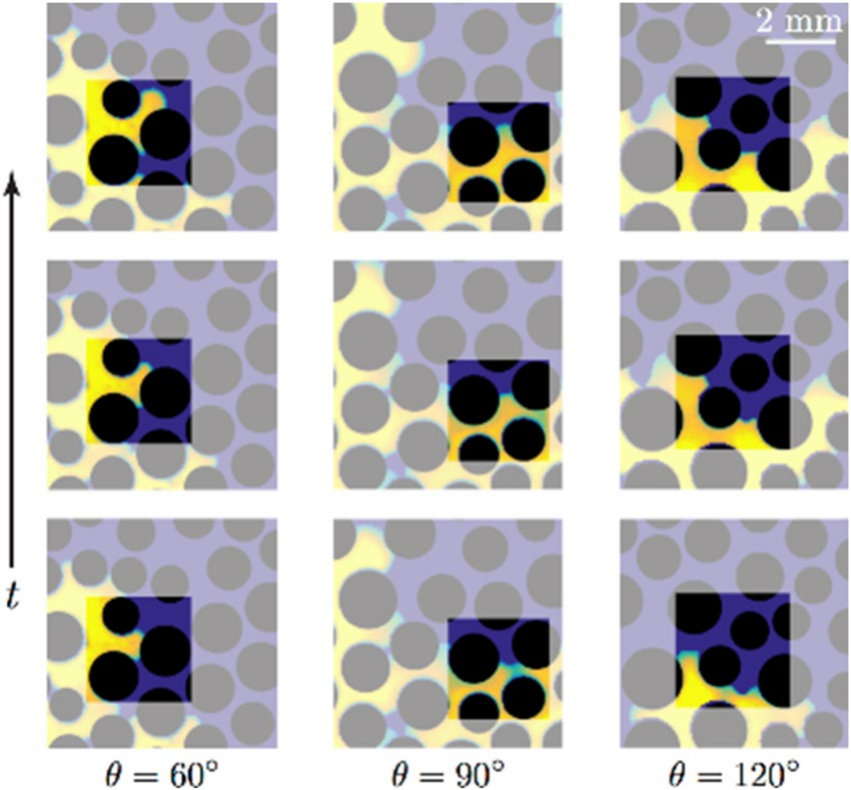
Experimental sequences of snapshots as the invading fluid traverses through a converging-diverging pore throat under different wettability conditions (left to right: *θ* = 60°, 90°, 120°). The experiments were conducted in a quasi-2D micromodel patterned with cicular posts (black) at low Ca, such that capillary forces dominate viscous forces. Here, the invading fluid (yellow) is water and the defending fluid (blue) is silicone oil. For *θ* = 90° (center column), the fluid-fluid interface curvature reverses from being convex in the converging section of the pore throat to being concave in the diverging section of the pore throat. In contrast, the fluid-fluid interface remains concave for *θ* = 60° (left column) and convex for *θ* = 120° (right column) through the invasion sequence.

To confirm these predictions, we investigate, with laboratory experiments, the impact of wettability on fluid-fluid displacement in micromodels patterned with circular posts. While circular posts constitute a much more simplified representation of a natural porous medium compared to the simulation domain extracted from a sand pack, the pore space between neighboring posts exhibits distinct converging and diverging sections. Here, we provide direct experimental evidence of the co-existence of concave and convex interfaces, as well as interface curvature reversal from converging sections to diverging sections of a pore-throat (Fig. 5). The effect of pore-geometry controlled local apparent wettability is most pronounced under intermediate wettability conditions (*θ* = 90°), where the invading fluid advances via drainage (convex interface) in the converging section of the pore throat, and imbibes (concave interface) in the diverging section of the pore throat (Fig. 5, center column).

## Apparent Wettability Number In Porous Media

Our direct numerical simulations of fluid-fluid displacement in a micromodel suggest that modeling two-phase flow in porous media by means of classical pore network models of uniform capillaries^18,19,21,35–38^ may obscure the nature of interfacial displacement, especially under intermediate wettability conditions. The converging-diverging channel provides a conceptualization of throats in porous media that may be a better representation of natural porous media. While we acknowledge the intrinsic disorder and irregularity of natural porous media, we explore how the converging-diverging angle *β* may impact apparent wetting properties in the form of a dimensionless number that accounts for surface chemical properties and pore geometry. This dimensionless quantity would render a simple but effective diagnostic tool to describe the apparent wetting behaviour of a porous medium.

In Eq. (3), the sign of *p*_*c*_ is controlled by cos(*θ* ± *β*). The trigonometric identity $$\cos (\theta \pm \beta )=\,\cos \,\theta \,\cos \,\beta \,\mp $$$$\sin \,\theta \,\sin \,\beta =\frac{\cos \,\theta \,\cos \,\beta }{\sin \,\theta \,\sin \,\beta }\mp 1$$, suggests that we define the *apparent wettability number*

4 $$W\,:\,=\frac{\cos \,\theta \,\cos \,\beta }{\sin \,\theta \,\sin \,\beta }=\frac{1}{\tan \,\theta }\frac{1}{\tan \,\beta }.$$

This quantity describes the relative importance of *θ* over *β* in controlling the interface shape. When $|W|\gg 1$, the sign of the interface curvature is controlled by *θ* alone—this is the case for strongly wetting (*θ* ≈ 0) or strongly nonwetting conditions (*θ* ≈ 180°). In contrast, when $|W|\ll 1$, the sign of the interface curvature is modulated by *β*—this occurs for neutrally wetting conditions (*θ* ≈ 90°). Combinations of *θ* and *β* such that |*W*| ~ 1 correspond to scenarios for which the interface is flat at either the converging or the diverging section of the pore.

We are interested in expressing *W* in terms of characteristic quantities describing the pore-space geometry in porous media. Assimilating the transition from a pore body (radius *r*_*b*_) to a pore throat (radius *r*_*t*_) as a converging channel, and assuming that the length of the channel is equal to the pore-body radius, we have tan *β* = (*r*_*b*_ − *r*_*t*_)/*r*_*b*_. Futhermore, assuming a relatively uniform, densely packed porous medium, we take the typical pore body as the space between four grains, so that the number of pore bodies *n*_*p*_ ~ *N* and the number of pore throats *n*_*t*_ ~ 2*N*, where *N* is the number of solid particles (grains). These assumptions lead to a relationship between *r*_*t*_ and *r*_*b*_, ${r}_{t}=\sqrt{\frac{\varphi A}{2\pi N}-\frac{{r}_{b}^{2}}{2}}$, where *ϕ* and *A* are porosity and total area of the porous medium (per unit depth), respectively. Substituting the mathematical expressions for tan *β* and *r*_*t*_ in Eq. (4), and taking the pore radius *r*_*p*_ = *r*_*b*_, we arrive at:

5 $$W=\frac{{r}_{p}\,{\cot }(\theta )}{{r}_{p}-\sqrt{\frac{\varphi A}{2\pi N}-\frac{{r}_{p}^{2}}{2}}}$$

**Figure 6:**
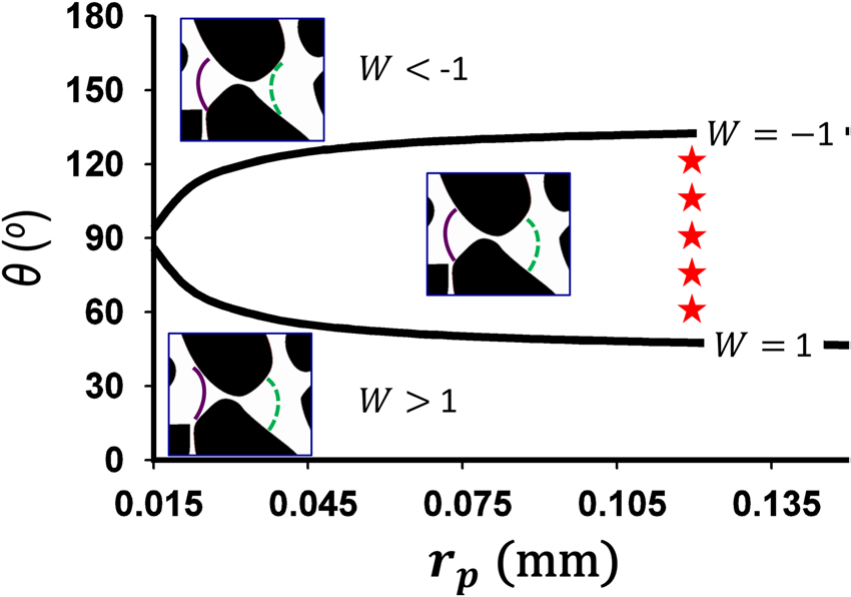
Phase diagram in *θ*–*r*_*p*_ space for the micromodel geometry with *ϕ* = 0.42, *A* = 94 mm^2^ and *N* = 222, separating the region where apparent wettability is affected by pore geometry (−1 ≤ *W* ≤ 1) from the regions where pore geometry influence on apparent wettability is negligible, leading to strict imbibition (*W* < −1) or strict drainage (*W* > 1) conditions. The boundaries (black solid lines) of the phase diagram correspond to the values of *W* = −1 and *W* = 1, where pore geometry and surface properties have equal impact on apparent wettability. The insets illustrate the shape of the fluid-fluid interface in converging (solid purple) and diverging (dotted green) sections of a typical pore throat. The flow direction for the insets in from left to right. The symbols (red stars) indicate the range of *θ* values where co-existence of concave and convex interfaces is observed in the numerical simulations (Figs 3 and 4).

In our simulation, *r*_*p*_ = 0.12 mm, *ϕ* = 0.42, *A* = 94 mm^2^ and *N* = 222. Using Eq. (5) we delineate, on a phase diagram of *r*_*p*_ − *θ*, the region of pore-geometry controlled apparent wetting (where co-existence of concave and convex interfaces is observed, corresponding to −1 ≤ *W* ≤ 1), from the regions where apparent wetting corresponds to strict drainage (*W* > 1) or strict imbibition (*W* < −1) conditions (Fig. 6).

For the micromodel pattern used in our simulation (*r*_*p*_ = 0.12 mm), the apparent wettability number *W* predicts that the co-existence of concave and convex interfaces to occur for *θ* between 48° and 132°. This prediction is consistent with our direct numerical simulation results, which shows the co-existence of concave and convex interfaces for *θ* between 60° and 120° (Fig. 6).

## Summary and Conclusion

We have studied the evolution of fluid-fluid interfaces through angular pore spaces under partial wetting conditions. Using direct numerical simulation, we show that the fluid-fluid interface is transformed from concave to convex in a single converging-diverging capillary under intermediate wetting conditions. We demonstrate that the macroscopic manifestation of the observed interface transformation in the converging-diverging capillary leads to the co-existence of concave and convex interfaces in a porous medium. We provide experimental evidence of this phenomenon in fluid-fluid displacement experiments in a micromodel.

Consistent with past studies, our results elucidate the interplay between pore geometry and contact angle *θ* in determining the sign of interface curvature (i.e., apparent wettability). We show that while apparent wettability is a function of *θ* alone under strong wetting conditions (as indicated by the classical Young’s law), pore geometry plays a crucial role in governing the apparent wettability under intermediate-wet conditions, which leads to co-existence of concave and convex interfaces. In the case of a converging-diverging capillary tube, the critical angles delineating the regime of apparent mixed wetting have been determined theoretically and shown in in Fig. 2(c) for a range of converging-diverging angles *β*. For porous media, the critical transition contact angles have been quantified via numerical simulations (Figs 3 and 4). In our example, we find values of the critical contact angle of 48° for the transition from drainage to mixed apparent wetting, and 132° for the transition from mixed apparent wetting to imbibition.

To capture the coupled effect of pore geometry and contact angle on apparent wettability, we derive the apparent wettability number *W* that estimates the range of *θ* values where pore geometry exerts a fundamental influence on the wettability of porous media and, likely, on the macroscopic displacement processes it mediates, such as enhanced oil recovery^39–41^ and geologic carbon sequestration^42,43^.

## Electronic supplementary material

Supplementary information

## Data Availability

The data presented in this manuscript will be available freely via sending a request to the corresponding author.
