# Supplementary material for: Pore geometry control of apparent wetting in porous media
Source: Sci Rep. 2018 Oct 24;8:15729. doi: 10.1038/s41598-018-34146-8 (PMC6200799; doi:10.1038/s41598-018-34146-8)
Supplement: Supplementary file 1 — Supplementary information [file 41598_2018_34146_MOESM1_ESM.docx]

**Pore geometry control of apparent wetting in porous media**

Harris Sajjad Rabbani^1^, Benzhong Zhao^2^, Ruben Juanes^3,4^, and Nima Shokri*^1^

1. School of Chemical Engineering and Analytical Science, The University of Manchester, Manchester, M13 9PL, United Kingdom
2. Department of Mechanical and Industrial Engineering, University of Toronto, Toronto, Ontario, Canada
3. Department of Civil and Environmental Engineering, Massachusetts Institute of Technology, Cambridge, Massachusetts, USA
4. Department of Earth, Atmospheric and Planetary Sciences, Massachusetts Institute of Technology, Cambridge, Massachusetts, USA

**Supplementary Information**

**
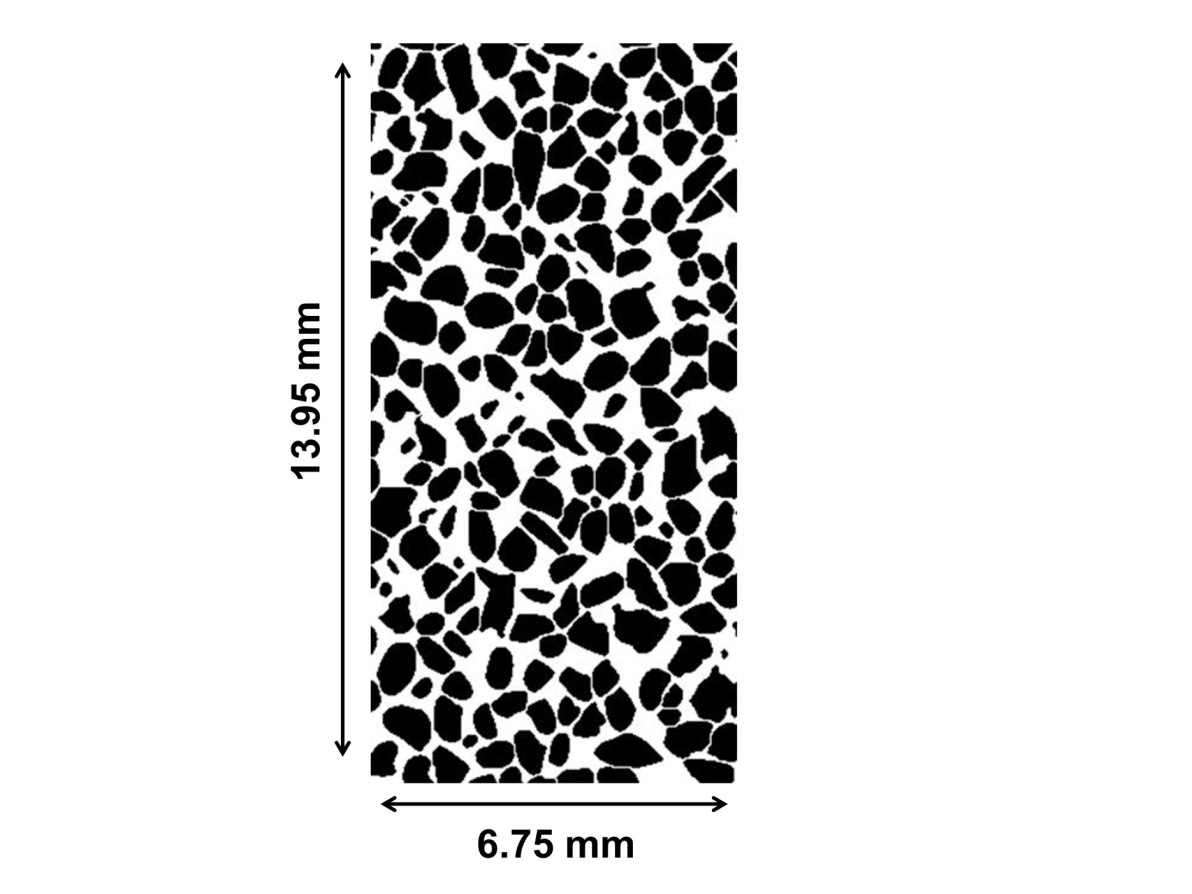
**

**FIG. S1.** The porous medium used for the numerical simulations, showing the grains (black) and the pore space available for fluid flow (white).
